# Forest structure, plants, arthropods, scale, or birds’ functional groups: What key factor are forest birds responding to?

**DOI:** 10.1371/journal.pone.0304421

**Published:** 2024-05-31

**Authors:** Swen C. Renner, Martin M. Gossner, Manfred Ayasse, Stefan Böhm, Miriam Teuscher, Wolfgang W. Weisser, Kirsten Jung

**Affiliations:** 1 Ornithology, Natural History Museum Vienna, Vienna, Austria; 2 Forest Entomology, Research Unit Forest Health and Biotic Interactions, Swiss Federal Research Institute WSL, Birmensdorf, Switzerland; 3 Department of Environmental Systems Science, Institute of Terrestrial Ecosystems, ETH Zurich, Zurich, Switzerland; 4 Institute of Evolutionary Ecology and Conservation Genomics, University of Ulm, Ulm, Germany; 5 Centre of Biodiversity and Sustainable Land-use, University of Göttingen, Göttingen, Germany; 6 Terrestrial Ecology, TUM, Freising, Germany; Southeastern Louisiana University, UNITED STATES

## Abstract

Forest birds respond to a diverse set of environmental factors, including those altered by forest management intensity, such as resource and habitat availability in the form of food or nesting sites. Although resource/habitat availability and bird traits likely mediate responses of bird diversity to global change drivers, no study has assessed the direct and indirect effects of changes in forest management and traits on bird assemblages jointly at large spatial scales. In this context the questions remain whether (1) the birds’ response to forest management changes through alterations in structural properties and/or food availability, or (2) if birds’ eco-morphological traits act as environmental filters in response to environmental factors. We audio-visually recorded birds at 150 forest plots in three regions of Germany and assessed the forest structure (LiDAR) as well as the diversity of the herbaceous layer and diversity and biomass of arthropods. We further assessed eco-morphological traits of the birds and tested if effects on bird assemblages are mediated by changes in eco-morphological traits’ composition. We found that abundance and species numbers of birds are explained best by models including the major environmental factors, forest structure, plants, and arthropods. Eco-morphological traits only increased model fit for indirect effects on abundance of birds. We found minor differences between the three regions in Germany, indicating spatial congruency of the processes at the local and regional scale. Our results suggest that most birds are not specialized on a particular food type, but that the size, diversity and species composition of arthropods are important. Our findings question the general view that bird traits adapt to the resources available.

## Introduction

Global change is affecting biological diversity with far-reaching consequences for ecosystem functioning and ultimately human wellbeing [1]. Despite extensive research on how biodiversity responds to global change [2], ongoing debates persist over the mechanisms underlying such responses. Assessing the relative importance of mediating roles of diverse environmental factors as filters remains unresolved. Diversity changes and abundance-shifts are caused by various types of environmental change resulting from different global change scenarios such as land-use change and intensification of land-use management [3]. These shifts can be augmented by eco-morphological trait-based filters. The traits can additionally alter community composition based on species-specific characteristics [4]. However, whether eco-morphological traits operate as environmental filters in these habitats (along with food availability), or if the trait-variability in birds is only driven by food availability (i.e. traits vary independent of the habitat) remains unresolved.

Land-use and forest management, influencing forest types and forest structural properties, leads to altered species assemblages and species responses, potentially favoring those traits adapted to local habitat conditions and impacting species’ vulnerability to structural changes [5]. Consequently, this sorting process—or environmental filtering—can lead to a loss of regional biodiversity as forest management continues to intensify. Environmental filtering can be seconded by species interactions or resource availability, that can further shape these assemblages [6]. Consequently, the effects of trait variability should be assessed along with species compositional changes, as both may be reasons for bird responses to change in forest management [7].

In this context, scale-dependencies matter, because increased landscape heterogeneity is associated with higher resources and niche availability and consequently with an increase in species numbers. However, local and landscape scale factors do not affect all birds in the same way [8]. Particularly, the role of eco-morphological traits on species composition, abundance and species diversity at different scales remains unresolved. To elucidate the mechanisms underlying the shifts in species abundances and composition across scales and forest management regimes, it is essential to understand the combined effects of direct and indirect pathways [2]. Bird species numbers and diversity are generally influenced by habitat structure, species-specific traits, seasonality, long-term turnover, and resource availability [9–15], and the impacts are evident at multiple spatial scales [16]. Intensified forest management and forest use alters resource availability such as food for the birds. For instance, altered insect biomass potentially impacts insectivorous bird populations [17]. Direct effects, involving factors like nesting sites, influence local bird occurrence [18]. Indirect effects, sometimes mediated through eco-morphological traits like bill size, could be shaped by habitat types, which in turn are influenced by forest management strategies [18]. Eco-morphological traits might mediate bird responses to management, influencing species numbers and abundances [19–21], while interactions between species can further shape these patterns [22]. However, the extent to which bird diversity responds directly vs. indirectly to environmental gradients and the detailed pathways of trait-variation remains uncertain.

Birds’ responses to forest management according to their eco-morphological traits can occur at individual, species, and assemblage levels [23, 24]. Traits, such as for instance bill size, limit a bird species’ capacity to consume insects. For instance, the size of the bill-opening and prey size need to match. Forest structure significantly influences bird species and abundance [23, 25–30], with different species favoring specific structures related to foraging strategies and eco-morphological traits [23, 28]. Thus, eco-morphological traits offer insights into how birds respond to arthropods as food availability, which in turn respond in biomass and species numbers to specific forest management measures. Evaluating resource availability, eco-morphological traits, and habitat structure featured in a single statistical framework will help to explain bird responses to land management.

**Fig 1:**
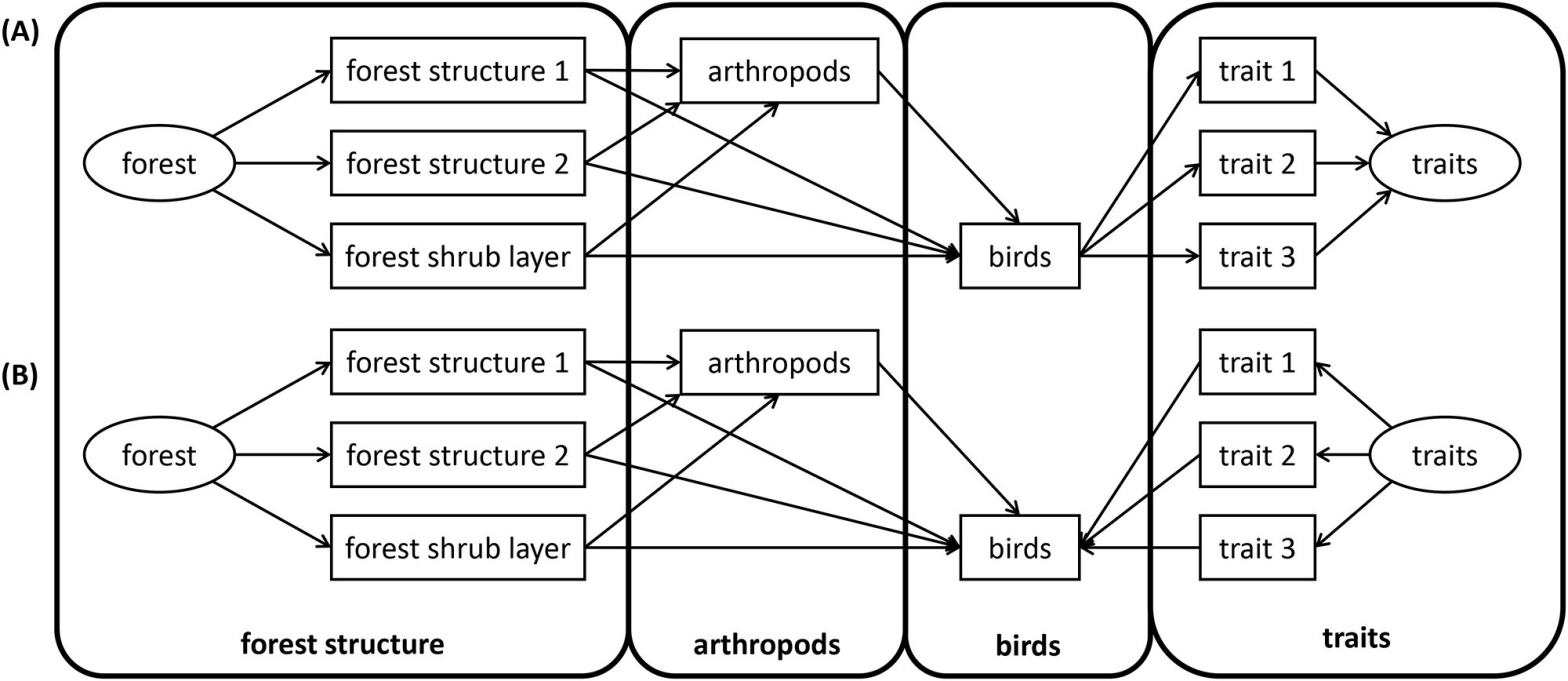
**(A)** Global model structure for Structural Equation Model, reflecting Hypothesis 1 (full model illustrated in S1 Fig). **(B)** Global model structure for Structural Equation Model, reflecting Hypothesis 2 (full model illustrated in S2 Fig). *Double-headed* or bidirectional arrows indicate variance or covariance. Latent variables (*ellipse*) are unobserved variables. *Ecomorphological traits* (S1 Table), arthropods (S2 Table), *forest structure* (S3 Table), and forest shrub layers (S3 Table) are hypothetical examples in this figure, and have been replaced by a set of measured parameters as listed in the corresponding S1 to S3 Tables.

The objective of our study is to examine how bird abundance is affected by environmental factors, specifically forest structural parameters (quantified by LiDAR—Light Detection and Ranging) and resource availability (arthropods, shrub and herbs), across local and regional scales in Germany. Particularly we focus on the role of eco-morphological traits, such as wing size, bill size, and tarsus size, in mediating bird responses to forest management by using Structural Equation Modeling (SEM). SEM offers insights into direct and indirect drivers [31], helping us understand potential mechanisms underlying relationships between forest management and bird assemblages. We explore two main pathways, representing two hypotheses: (1) forest structure and resource availability affect bird traits and diversity, while eco-morphological traits are of limited importance in sorting the bird community, but are affected by the sorting of species and their abundance through food availability (Fig 1A illustrates the general pathways for this model structure)–we name this hypothesis "Habitat-food filter hypothesis;" vs. (2) forest structure and resources influence bird assemblages via trait-mediated mechanisms, reflecting environmental filtering of traits (Fig 1B illustrates the general pathway for this model structure)–we name this hypothesis "eco-morphological filter hypothesis."

## Study area and methods

### Study area

Our study is part of the large-scale and long-term research platform ’Biodiversity Exploratories’. A detailed description of the study area, selection of plots and classification procedures is given in Fischer, Bossdorf [32], which is summarized here: The Biodiversity Exploratories project encompasses diverse grassland and forest plots across three regions in Germany, covering a total area of approximately 422 sq-km in Schwäbische Alb, 1,300 sq-km in Schorfheide-Chorin, and 1,300 sq-km in Hainich-Dün. The three regions are located in the Biosphere Reserve Schwäbische Alb plateau in the Southwest (approximate center coordinates: 48.41° North, 9.50° East, 500–800 m), the Hainich-Dün area including the National Park in the Center (51.13° North, 10.40° East, 285–550 m), and the Biosphere reserve Schorfheide-Chorin (53.02° North, 13.88° East, 3–140 m) in the Northeast of Germany. The three regions differ in climatic, geological and topographical conditions, with mean annual temperatures ranging from 6.0–8.5 C (Southwest 6.0–7.0 C, Center 6.5–8.0 C, Northeast 8.0–8.5 C) and mean annual precipitation from 500–1000 mm (Southwest 700–1000 mm, Center 500–800 mm, Northeast 500–600 mm) [32]. The plots are managed under varying intensities to facilitate detailed ecological studies.

The forests included into the study design of the Exploratories cover a total of 500 hectares in each region. Each region covers various forest types and management practices across three German regions: In Schwäbische Alb, the plots include beech, spruce, and oak forests, managed as age class forests, selection forests, and unmanaged forests. Schorfheide-Chorin features beech, pine, oak, and spruce forests, with management practices including age class forests (both pure pine stands and pine stands mixed with beech) and unmanaged forests. Lastly, Hainich-Dün focuses on beech and spruce forests, managed as age class forests, selection forests, and unmanaged forests.

The Biodiversity Exploratories cover a total of 150 forest plots in the three regions of Germany, with 50 plots in each region. The forest plots in the Biodiversity Exploratories project have been implemented through a stratified random sampling method, ensuring a diverse representation of forest types and management practices, and are marked and monitored with precise geographic and environmental data collection protocols.

Forest structure and management regimes were assessed at each forest plot of 100 m by 100 m within a larger forest management unit (i.e. the plots are embedded within a forest stand, which is larger than our study plot; the size of the management units were 4 ha to 8 ha, and the plot was always installed in the center), including a 30-m buffer [32]. Within this 160 m by 160 m area, the forest is of one management type and the structure is largely homogeneous; however, beyond the buffer, it is possible that a forest edge or frequent changes in land use management and land cover occur.

### Sampling of organisms and trait assessment

All taxa (birds, arthropods, plants) were surveyed within the boundaries of the forest plot to guarantee spatial congruency. All data sets were compiled in a way to guarantee a maximum temporal overlap of sampling periods.

**Birds** were surveyed using fixed-radius audio-visual point-counts. Bird surveys were standardized by time and space to avoid the need for rarefaction or abundance extrapolations. Each region featured 50 point-count localities in forests, with each plot represented by a single point-count in the center of the plot. Specifically, we recorded all birds exhibiting territorial behavior (singing and calling) for five minutes per plot and time period. Each plot was visited five times between March, April, twice in May, and June 2008. By doing so, we assessed the abundance of bird individuals at the plot level.

A minimum of five and a maximum of 15 plots per day were surveyed by one observer from sunrise to 11:00 h. The evening chorus was surveyed after 17:00 h to sunset once. In general, the sequence of visited plots was randomized. Observers were the same within each region during the season to minimize observer bias. The maximum number of birds displaying plot^-1^ year^-1^ (i.e. the maximum record of individuals per species over the five repetitions) was used as a measure for abundance of birds at a given forest stand. We considered a species as present at any given plot if recorded at least once during the survey period and used the number of all birds present as a measurement of bird species number. Further details on the birds’ monitoring and methods as well as the standardization procedures are detailed in Renner, Baur [15] and Renner, Suarez-Rubio [23].

We derived ecological and morphological traits for each bird species [further details in 33]. For 56 bird species we measured bill length, bill width, tarsus length, wing length and length of 3^rd^ primary in the same study plots as we did for all bird species in the year 2014/2015 [33]. Specifically, we measured bill height at the proximal end of the operculum, bill length from the tip to the proximal end of the operculum, tarsus length from the intertarsal joint to the bent toes [34], and bill width at the widest point of the operculum (details on species and traits in S1 Table). Each metric was documented as a mean value with standard deviation (SD), and the range specified by the minimum and maximum measurements. These morphometric data were collected from our own extensive field studies [33]. All measurements were taken in millimeters (mm) to the nearest of 0.1 mm, ensuring precision and to facilitate comparison with existing literature. For species for which we do not possess our own measurements of eco-morphological traits, we amended the information as best as possible from publications [as referenced and summerized in 33]. The measurable traits have been averaged per species and added to our data framework. In the assessment of morphometric traits, we conducted an analysis to ensure reliability and consistency. All traits were scrutinized for collinearity, with a threshold set where the coefficient of determination |r^2^| > 0.7, indicating high correlation. Consequently, we chose the maximum values of the eco-morphological trait parameters. The set of chosen eco-morphological traits has low or absent collinearity (S1 Table).

We also classified each species based on the major nutritional intake during the breeding season into four feeding groups: insectivore, granivore, omnivore (i.e. no specific preference known, or more than one out of the other categories), and carnivore (i.e. non-insectivore carnivores) bird species. To understand the response of each of the four major feeding groups, we treated the groups carnivore, granivore, insectivore, and omnivore birds by grouping factors in the analysis (details of analysis explained below).

**Arthropods’** abundance was assessed using stratified trap counts. In the canopy (between 5 m and 27 m height depending on the forest stand age) and understory (1.5 m) composite flight interception traps [35] and for epigaeic arthropods pitfall traps (funnel traps of 16 cm diameter; our general trap design followed Lange, Gossner [36]) were used. We added a 3% copper-sulfate solution and a drop of detergent to reduce surface tension as sampling fluid. We sampled traps at monthly intervals between March and October, largely overlapping the periods of the bird surveys. Arthropods were transferred to 70% ethanol in the field and sorted to order level in the laboratory. We pooled all arthropods sampled in each stratum during each sampling interval for further analysis. We excluded one plot (HEW34) from analysis with SEM models, because pitfall traps at this particular plot were run for a limited time due to disturbance by large mammal activities and would thus not allow a full estimate of species number and abundance of arthropods. To estimate arthropod abundance and species number per forest plot we used groups as listed in ’Selection 1’ of S2 Table.

In addition, we tested if replacing ’arthropod abundance’ by ’arthropod biomass’ would improve model fit, because birds select prey more likely by mass or size and less likely by prey species. We included the taxonomic groups of Coleoptera and Hemiptera, Auchenorrhyncha and Heteroptera in the arthropods biomass analysis, which were identified to species level, using allometric relationships (for details, see Seibold, Gossner [37]). We estimated the biomass of these insects by applying a general power function published by Rogers, Hinds [38] based on species-level information on body length from literature [39]: biomass (in g) = 0.305 × L2.62/1,000 where L is the mean body length of a species in millimeters.

Coleoptera and Hemiptera, Auchenorrhyncha and Heteroptera are known to be valuable food resources for many birds. However, model fit did not change in outcome when biomass was used instead of arthropod abundance, and showed only minute differences in path-estimates (’bird.abm.n2’, *p* = 1.000, Goodness-of-fit = 1.000, AICc = 0.290). Consequently, we continued with arthropods abundance throughout all models.

**Plants** were recorded at all plots in spring and in late summer in an area of 20 m by 20 m [40]. We identified all vascular plants and estimated the percentage cover per species separately for two tree-layers (5–10 m and > 10 m), the shrub layer (0 m to 5 m), and the herbaceous layer (including phanerophyte seedlings). To assess the total species number per plot, we combined the spring and summer records.

### Forest structural parameters

Quantifying forest structure is challenging [23, 41, 42], but Light Detection and Ranging (LiDAR) provides a quantitative approach [26, 23, 43–45], offering valuable insights into the impact of management on local structure and bird diversity [26, 43]. To obtain continuous environmental variables related to forest structure, all forest plots were scanned during foliated season in 2008 (Center), 2009 (Northeastern) and 2010 (Southwestern), using airborne LiDAR. No noteworthy forest management actions took place between 2008 to 2010 and thus we are confident that forest structure obtained by these LiDAR scans represents the existing forest structure during the taxonomic surveys of plants and animals within each forest stand. All plots were scanned at a flight altitude of 400 m, using a Riegl LMS-Q 560 scanner operated by MILAN GmbH [45].

The scanner operated at a pulse repetition rate of 240 kHz with a nominal point density of 22 m^−2^ to 106 m^−2^ and recorded up to seven peaks in the intensity of laser pulse returns. Point density varied up to 1,159 m^−2^, depending on flight velocity and reflectivity. We down sampled the point density to a maximum of 500 m^−2^ to get more homogeneous LiDAR variable conditions at the plots. The footprint diameter ranged between 20 cm to 30 cm. The sampling accuracy resulted in 50 cm horizontal and 15 cm vertically. We calculated 31 parameters for the inner and outer forest structures of each 100 m by 100 m plots (mean ± s. d.). We calculated all structural parameters from normalized baseline (raw) data and the canopy height model.

We used Random Forest algorithms [46, 47] with 100,000 randomizations implemented in R to select the five most important structural parameters from the total set of available LiDAR variables for bird species presence/abundance (details on LiDAR variables in S3 Table). To assess structural parameter importance we sorted the parameters for each species according to its percentage of Mean-Square-Error-values (%IncMSE) [47] and selected the highest ranked variables for further analysis; from those variables, we further excluded all auto-correlated variables with |r^2^| > 0.7 yielding a set of LiDAR forest variables for birds with lowest auto-correlation; the best choice according to RandomForest was Open Stem Zone, Vertical Variation, Variation in Forest Height (Forest height ± SD), Canopy Height, and the Regeneration-layer [45] (S3 Table); these parameters represent structural differences of managed forests within the three regions [45].

### Statistical analysis

To assess the most important environmental drivers for birds’ abundance and species numbers, we used Structural Equations Modeling (SEM). Abundance per species has shown the best fit in many other studies, while species richness or absolute abundance performed equally. Abundance data reflect the relative importance of each species in the assemblage within our study regions. Collinearity analysis of our data showed high correlation between the parameters (|r^2^| > 0.7) and consequently we focused on abundance of birds as the response variable.

In our study, we developed a series of Structural Equation Models (SEMs) [31, 48] through an iterative process to test our two competing hypotheses. Initially, we constructed a comprehensive model incorporating all potential pathways, as delineated by Hypothesis 1, and contrasted it with a model based on Hypothesis 2. The models’ adequacy was evaluated using several fit indices, including the model Chi-squared (χ^2^-statistics, with a desired *p*-value > 0.05), the Goodness-of-Fit (GFI > 0.95), and the Root Mean Square Error of Approximation (RMSEA < 0.08), along with their respective significance levels and thresholds. Subsequently, we refined the models by removing highly correlated parameters and those deemed less relevant for bird abundance. This led to a reduction of LiDAR parameters from ten to three that best represented the forest structure, i.e. ’Vertical Variation [entropy], Canopy Height [q9], and the Regeneration-layer [RegenerA]’ (S3 Table). For piecewise SEM we focus mainly on the Fisher’s C test to asses global versus local models.

Further simplification involved eliminating statistically insignificant pathways, ensuring such exclusions did not impair the overall fit of the model. Adjustments included the modification or removal of traits and forest variables, and alterations of paths or additions of "missing paths," aimed at improving the overall models’ predictive power. These modifications were made while testing the null hypothesis of no relationship between bird abundance and other variables, ending in a refined global model structure presented in Fig 1A and 1B for Hypotheses 1 and 2, respectively. We added a comparative analysis between the global full and local (best fitted) models using ANOVA (lavTestLRT), evaluating their relative efficacy. Additionally, the influence of arthropods as a primary food source was examined by their exclusion from the SEMs and observing the impact on the fit indices and Akaike’s Information Criterion (ΔAIC).

Finally, we introduced grouping parameters based on regions and functional groups, treating these as non-numeric factors within the SEM framework to explore potential regional and functional differences. This approach generated three distinct full models and alternative models for each group, which are shown in S3 Fig for the alternative models.

Latent variables are variables that are unobserved, but whose influence can be summarized through one or more indicator variables [48]. Latent variables are useful for capturing complex or conceptual properties of a system that are difficult to quantify or measure directly. We used ’Traits’, ’Arthropods’, ’Birds’, ’Forest’ and ’Plants’ as latent variables; if latent variables decreased overall model fit we subsequently omitted these. The used latent variables are at the meta level, for example the latent variable ’Forest’ represents the forest types as characterized by the LiDAR parameters in the respective SEM.

In total we calculated 329 SEMs with the outlined two main paths (Fig 1); 260 SEM out of these converged, 214 were rejected based on *p* of the χ^2^ statistics, leaving 46 that were retained with varying levels of fit in *p* of χ^2^ and ΔAIC.

We used the packages RandomForest [47], SEM [48], and lavaan [49] in R [50].

### Ethical statement

Capturing of animals and handling of plants were performed in compliance with laws and regulations of German federal and state legislation. All permits to access protected areas, capturing/handling of species/protected species, and handling wildlife were granted by the *Regierungspräsidium Tübingen* for the Schwäbische Alb, by *Thüringer Landesamt für Verbraucherschutz* for the Hainich-Dün, and by the *Landesamt für Umwelt*, *Gesundheit und Verbraucherschutz Potsdam* for the Schorfheide-Chorin. All land owners and land-users approved access to their areas prior to the study.

## Results

Abundance of birds was strongly related to forest structure (LiDAR), plants, and arthropod diversity. The piecewise SEM analysis revealed that the initial X2-SEM (Hypothesis 2: eco-morphological filter hypothesis) demonstrates a better global fit to the data than the Y1 model (Hypotheses 1: Habitat-food filter hypothesis; Table 1, compare all model summaries of the piecewise full SEM in S5 Table). Hypothesis 2 is supported by the goodness-of-fit tests (X2: χ^2^ = 6.07, *p* = 0.53, df = 7; Y1: χ^2^ = 19880.33, *p* ≤ 0.01, df = 112). The Fisher’s C test supports X2’s adequacy (X2: Fisher’s C = 15.95, *p* = 0.32, df = 14), while Y1 shows a relatively poorer fit and should be rejected (Y1: Fisher’s C = 2302.89, *p* ≤ 0.01, df = 224). Despite this, Y1’s lower AIC suggests it may be more parsimonious (-7690.53 vs. X2’s -3522.61). Analysis of coefficients and significance levels across both models highlights key relationships, such as the significant negative effect of plants (’number_herbs’) on bird abundance in X2 and a similar negative impact on arthropods in Y1.

**Table 1:** Summary for Structural Equations Modeling (SEM) output of models that converged. Response variable is abundance in all cases. DF: degree of freedom, GFI: Goodness-of-fit-Index, SRMR: Standardized Root Mean Square Residual. All SEM summaries are listed in S4 Table. *Gray shaded* addressed Hypothesis 1 ("Habitat-food filter hypothesis"), all others Hypothesis 2 ("eco-morphological filter hypothesis"). Models sorted descending according to GFI.

Model name	Hypothesis	Arthropods	Groups	Model χ^2^	DF	*p* of χ^2^	GFI	SRMR	Figure
Outline for m1	H1	n/a	n/a	n/a	n/a	n/a	n/a	n/a	1A
Outline for m2	H2	n/a	n/a	n/a	n/a	n/a	n/a	n/a	1B
m2 *(best fitted)*	H2	included	not grouped	0.110	11	1.000	1.000	0.001	2B
m2.g3Expl	H2	included	3 regions	0.976	33	1.000	0.999	0.002	S3B
m3na.fg	H2	not included	4 functional	3243.630	68	≤0.001	0.998	0.158	n/a
m3na.g3Expl	H2	not included	3 regions	9329.320	51	≤0.001	0.998	0.139	n/a
m4na.g3Expl	H1	not included	3 regions	9849.317	48	≤0.001	0.990	0.779	n/a
m3na	H2	not included	not grouped	1374.671	17	≤0.001	0.956	0.043	n/a
X2 *(full model to m2)*	H2	included	not grouped	53360.347	159	≤0.001	0.639	0.135	S2
m1.fg	H1	included	4 functional	21208.679	76	≤0.001	0.628	0.918	n/a
m4na.fg	H1	not included	4 functional	21208.378	64	≤0.001	0.602	1.021	n/a
m1	H1	included	not grouped	6185.672	19	≤0.001	0.565	0.634	2A
m4na	H1	not included	not grouped	6185.509	16	≤0.001	0.536	0.702	n/a
m1.g3Expl	H1	included	3 regions	5719.697	57	≤0.001	0.549	0.692	S3A
Y1 *(full model to m1)*	H1	included	not grouped	103529.883	172	≤0.001	0.118	1.421	S1
m2.fg	H2	included	4 functional	Model did not converge (too low *N*)					n/a

Excluding forest structural or plant or arthropod parameters from any of the presented SEM, decreased model fit significantly. However, effects on abundance of birds was mediated by eco-morphological traits congruently in all models. The SEM testing our Hypothesis 1 performed less well than the models testing for Hypothesis 2. This emphasizes mediating effects via eco-morphological traits are in effect, suggesting that forest management alters bird assemblages via environmental filtering. Models improved by including arthropods (Table 1), indicating food availability as an important driver of bird assemblages.

**Fig 2:**
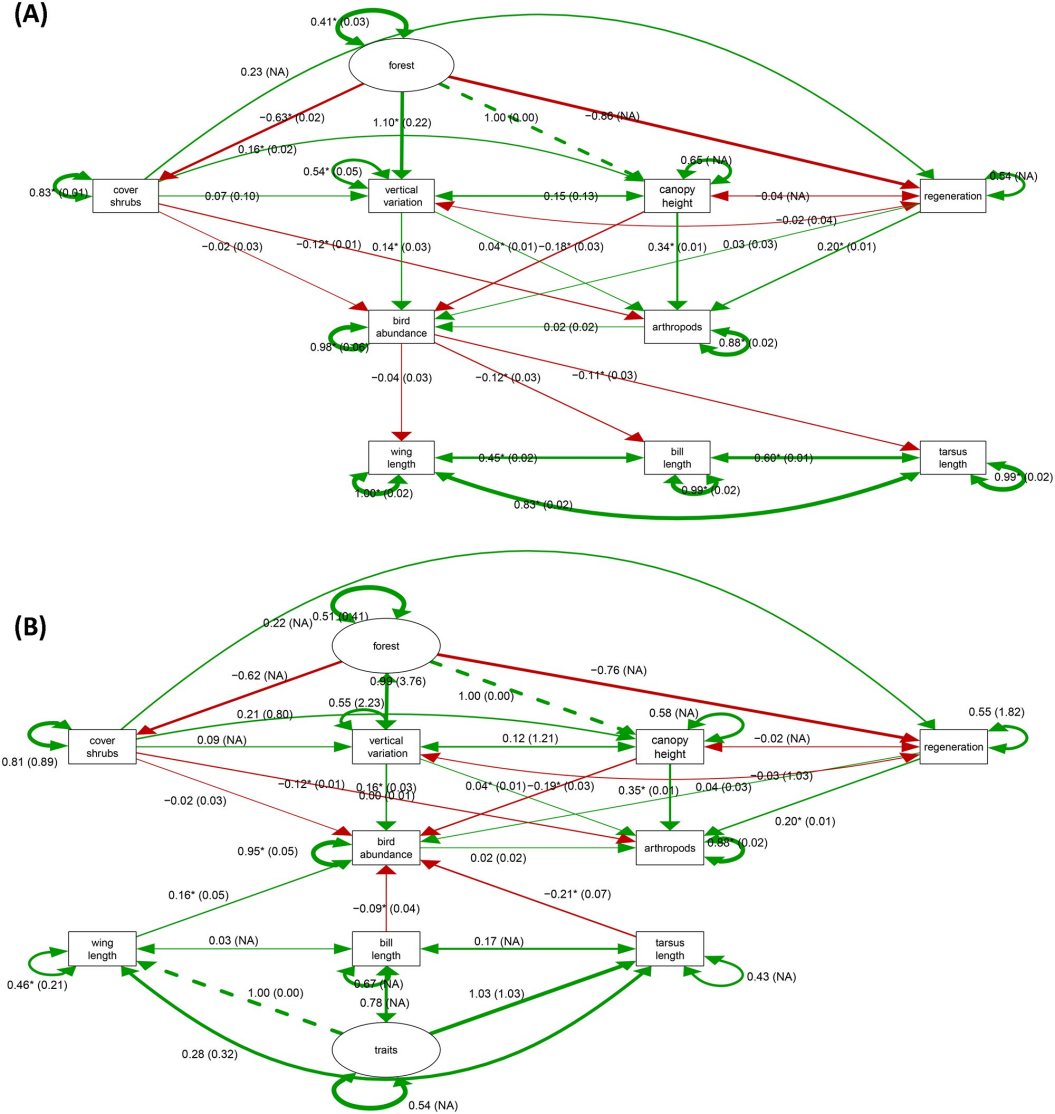
(A) Structural Equations Model "m1" reflecting Hypothesis 1 with arthropods, followed by (B) the best fitted Structural Equations Model "m2," reflecting Hypothesis 2. *Double-headed* or bidirectional arrows indicate variance or covariance. Latent variables (*ellipse*) are unobserved variables. *Red arrows* indicate negative vs. *green arrows* positive regression coefficient. Shown are standardized parameter estimates including (s.e.) in brackets; * *p* ≤ 0.05 (overall model parameters in Table 1).

The best fitted SEM followed the main path ’Forest → plants → birds ← traits’ and reflects Hypothesis 2, including arthropods (χ^2^ = 0.110, df = 11, *p* of χ^2^ = 1.000, GFI = 1.000, "m2" in Table 1; represented in Fig 2B, with the corresponding full model "X2" in S2 Fig). The best fitted SEM was followed by a set of very similar second-best-fitted models, which all included arthropods (Table 1), however had specific paths reduced or excluded. This set of the subsequent SEM, with paths exclude comparted to "m2" (Table 1), all confirm the general model-structure of "m2." This indicates that species abundance of birds is affected by habitat (LiDAR forest structural parameters), and food resources (arthropods, plants/seeds). The alternative hypothesis (Hypothesis 1; illustrated in Fig 2A, with the corresponding full model in S2 Fig) with the global structure of ’Forest → plants → birds → traits’ performed less well and most altered models of "m1" were rejected (χ^2^ = 6185.672, DF = 19, *p* of χ^2^ < 0.001, GFI = 0.565; Table 1).

Comparing the full SEM with the finally best-fitted-SEM resulted in significant better fit of the latter, for both hypotheses (lavTestLRT χ^2^ Difference Test: "m1" df = 19, χ^2^ = 6185.7, "Y1" df = 172, χ^2^ = 103529.9, *p* ≤ 0.001; "m2" df = 11, χ^2^ = 0.11, "X2" df = 159, χ^2^ = 53360.35, *p* ≤ 0.001).

Model results were largely consistent across the three regions (S3 Fig) with only one visible difference, i.e. in the central region, the paths from forest latent to regeneration is negative, while absent (n/a) in the northeast, and positive in the southwest. Nevertheless, the model structure following Hypothesis 2, ’forest → plants → arthropods → birds ← traits’ was better supported then the alternative Hypothesis 1 (Table 1).

Model results were somewhat inconsistent when grouped according to the four functional groups, because the overall functional group models did not perform as well as all other SEM (Table 1); these SEM were mostly rejected based on *p* of χ^2^ (Table 1). Nevertheless, the general pattern in terms of testing the major paths was consistent with the best fitted models without grouping, i.e. Hypothesis 1 (SEM "m4na.fg") rejected, and Hypothesis 2 ("m3na.fg") not rejected. The models divided into the four functional groups (carnivore, granivore, insectivore, and omnivore birds) showed globally consistent patterns, but differences in their detailed paths; effects from bird abundance to traits changed direction between groups in some cases or from forest structure to bird abundance. For instance, the paths "vertical variation" → "bird abundance" was negative for carnivores, but positive in all three other functional groups (S4 Fig).

Models with grouping factors including both regions and functional groups did not converge, because of too few *N* in several combinations. Hence, these models are not presented here.

## Discussion

Our study shows the significant roles of forest structure (a proxy for forest management), plant, and arthropod availability (proxies for nutritional resources) as drivers of bird diversity. However, the eco-morphological traits of birds had a comparatively greater effect on abundance than forest structure and nutritional resources. This outcome challenges the conventional view that bird traits, typically focusing on life-history, are adapted based on nutritional resources [21, 51]. Our findings suggest that most birds, while not necessarily specialized concerning their diet, are nevertheless limited to some extent on the size, diversity or species composition of arthropods and plants as food resources. The best-fitting model supports our "eco-morphological filter hypothesis" (Hypothesis 2), which postulated ’forest influences on plants, arthropods, and bird abundance, with traits affect bird abundance,’ suggesting that environmental filtering plays a significant role in shaping these relationships.

Regional differences in abundance and traits were observed but had minimal impact on our model outcomes. The lack of major discrepancies between regions contrasts with numerous studies that report significant local differences in bird responses to land-use changes [21]. The consistency across regions in our study could have several reasons, one of which may be the use of standardized data across the regions, coupled with a uniform analytical approach, a methodology not commonly employed in previous studies. Another reason is that several studies have an extended view of regional, hence partially cover a variety of biomes and different ecosystems, which can change the species composition. A major reason for the discrepancy between our study and other studies can be the scale and the habitats/ecosystems considered, hence discrepancies show a need to avoid extrapolation.

Shifts in abundance and changes in species numbers or diversity in response to global change drivers are well-documented in studies on bird assemblage [52–54]. For example, there are a plethora of studies enlightening aspects of turnover in species number/compositional changes of bird assemblage [13] in response to land-use, land cover [29], climate change [21, 55], forest fragmentation [56], selective logging [56], landscape composition [57], and urban development [58–61], with spatial distance [62, 63], dispersal abilities [64–66]), environmental [54], and stochastic aspects [67–69] being relevant for the observed relationships. However, integrating two or even several of these aspects within a single framework remains uncommon, despite the extensive existing research on birds. Our study extends these findings by showing that eco-morphological traits are critical for understanding variation in bird assemblages [23, 24, 70–73].

Functional or eco-morphological groups have been found to be important as mediators, filters, or drivers in diversity studies, explaining at least part of the variation in bird assemblages encountered. Some studies showed that life-history traits can explain modifications in species numbers or species assemblages of specialized taxa, and ultimately affect diversity [56]. As expected, all four main functional groups we classified are explained best by models which focus on the conceptual connection between food resource and major feeding class. For example, granivores increase with forest site complexity, characterized by having a richer herbaceous layer, because there are more relevant food items present for the birds. This is a similar finding with other studies, e.g. in Australia, where vegetation complexity was found as an important predictor of bird diversity [74]. For instance, taller vegetation provides more ecological niches and thus could harbor assemblages with higher species number and functional diversity [74]. In this case, resource use behavior was considered an important functional trait because it can link species to their resource base, e.g. granivores are linked to seeds, and this suggests niche partitioning in bird assemblages [74]. More complex vegetation provides larger ’ecological space’ with more resources, allowing the coexistence of more species with disproportionately more diverse foraging substrate used [74]. Similarly, insectivore birds are found in areas with higher abundance of prey, where insect-preying birds increase temporally and locally when insect abundance increased. In addition, plants can be a direct food resource for some bird species, but also indirectly through supporting higher arthropod abundance and diversity.

The diversity of forest-dependent birds can decrease with the homogenization and simplification of initially complex-structured vegetation, as found for many bird assemblages. For example, in the Atlantic forests of Brazil [57], higher amounts of forest edges are associated with higher bird species diversity, probably because the increasing interspersion/juxtaposition of different habitat types in landscapes with more forest edges can increase resource availability and foraging efficiency of non-forest-dependent birds [57]. We found a similar pattern in structurally richer forest due to increased vertical variation. In a study assessing biodiversity, including bird species number in the Black Forest (Southwest Germany), drivers for increasing diversity of organisms were mainly those having a rich structural diversity indicating a higher diversity and likely biomass of arthropods [75–77]. Diversifying the tree species composition in a forest management setting of Central Europe affects the forest diversity in manifold ways [78]: different tree species contribute directly to the species diversity because of, e.g. mammals’, birds’, or lichens’ dependency on the trees as resources [79]. In addition, tree species diversity increases forest structural diversity which supports increased availability of e.g. nutrients in form of arthropod prey and thereby indirectly promotes bird diversity [80]; our results confirm these findings, since the best fitted SEMs include arthropods and are slightly better fitting than SEM excluding the arthropods.

We used only one year of bird data because this allowed us to link bird communities to a unique dataset of plants, arthropods and forest structure. The spatiotemporal shifts in bird abundance and species composition can be considerable between years [13]. In birds, dynamic systems with a complex mix of stable and variable components occur and produce changes in species composition and abundance over various spatial and temporal scales [81]. However, species turnover within years is considered as ’low’ and between years as ’moderate’ in temperate areas [82–84]. This suggests that focusing on one year in which multitrophic and forest structural data is assessed simultaneously across spatial scales is of high relevance to explore mechanisms underlying the relationships between forest management and bird assemblages. By focusing on one year of data for birds, arthropods, and plants, we have eliminated confounding effects of year in our models and spatiotemporal compatibility of the data is maximized—an approach novel to bird assemblage studies.

In the context of interannual variability, stochasticity and random processes play a significant role [68], and predicting changes in assemblage composition without addressing random factors is prone to draw misleading conclusions [67–69, 85]. Deterministic processes such as those driven by habitat structure and heterogeneity [26], species-specific ecological traits [11, 33], seasonality [15, 86], or resource availability such as nutrition [82, 84], have been shown to be important in many ecosystems and for many taxonomic groups. However, understanding the random processes determining the species number, diversity, and abundance of organisms remains a key challenge. In a previous study based on a multi-year dataset of the same study regions [68], stochastic processes explain a substantial proportion of the species number and diversity of the bird assemblage [67, 68]. The random portion of the bird assemblage has been moderate for the Northeastern region (2008: 0.86, 2008–2012: 0.71; [68]) and relatively low for the Center (2008: 0.44, 2008–2012: 0.45; [68]) and Southwest (2008: 0.43, 2008–2012: 0.47; [68]). This, in turn, infers that there are indeed deterministic drivers explaining the bird assemblage in the three regions. The biggest obstacle to a better understanding of the random portion of drivers in assemblage-studies is that the datasets can be used to analyze either random factors OR deterministic processes, but there is currently no feasible way to understand both parts within the same common modelling framework [67, 69, 85].

## Conclusions

Our research adds to the growing body of evidence that while environmental factors play foundational roles in shaping bird assemblages, the mediating effects of eco-morphological traits are pivotal. While we observed differences between the three regions in Germany, our results suggest spatial congruency of the main determinants of bird species assemblages at both local and regional scales. Our findings challenge the prevailing assumption that bird traits adapt uniformly according to available resources. To comprehensively understand the adaptive responses of bird traits, their integration into a broader analytical framework is essential. Our study underscores the complexity of avian responses to alterations in forest management and highlights the necessity of incorporating eco-morphological traits, resource availability, and forest structural parameters in future research. Moreover, conservation organizations are encouraged to move beyond the limiting metrics of species diversity or abundance. A more detailed approach, utilizing sophisticated interaction networks, is important to elucidate the "conservation value" of specific forest management or habitat conservation strategies. Such a paradigm shift is crucial for a general understanding and effective stewardship of our forest ecosystems.

## Supporting information

S1 Fig Structural Equations Model "Y1" the global model to "m1" reflecting H1 with arthropods. *Double-headed* or bidirectional arrows indicate variance or covariance. Latent variables (*ellipse*) are unobserved variables. *Red arrows* indicate negative vs. *green arrows* positive regression coefficient. Shown are standardized parameter estimates including s.e.; ***: *p* ≤ 0.001; ** *p* ≤ 0.01; * *p* ≤ 0.05. (Model parameters in Table 1, and S4 Table).
(JPG)

S2 Fig Structural Equations Model "X2" the global model to "m2" reflecting H2 with arthropods. *Double-headed* or bidirectional arrows indicate variance or covariance. Latent variables (*ellipse*) are unobserved variables. *Red arrows* indicate negative vs. *green arrows* positive regression coefficients. Shown are standardized parameter estimates including s.e.; ***: *p* ≤ 0.001; ** *p* ≤ 0.01; * *p* ≤ 0.05. (Model parameters in Table 1 and S4 Table).
(JPG)

S3 Fig **(A)** Structural Equations Model "m1.fit.g3Expl" showing the SEM as for m1/H1, but grouped for the three regions. Shown are the SEM for each of the three Exploratories from left to right as: Schwäbische Alb (southwest), Hainich-Dün (center), and Schorfheide-Chorin (northeast). **(B)** Structural Equations Model "m2.fit.g3Expl" showing the SEM as for m2/H2, but grouped for the three regions (same sequence from left to right). *Double-headed* or bidirectional arrows indicate variance or covariance. Latent variables (*ellipse*) are unobserved variables. *Red arrows* indicate negative vs. *green arrows* positive regression coefficients. Shown are standardized parameter estimates (Model parameters in Table 1, and S4 Table).
(JPG)

S4 Fig **(A)** Structural Equations Model "m3na.fg" showing the SEM as for m1/H1, but grouped for four functional groups. Shown are the SEM for each of the four functional groups from left to right as: carnivore, insectivore, granivore, and omnivore. **(B)** Structural Equations Model "m4na.fg" showing the SEM as for m2/H2, but grouped for four functional groups (same sequence from left to right). *Double-headed* or bidirectional arrows indicate variance or covariance. Latent variables (*ellipse*) are unobserved variables. *Red arrows* indicate negative vs. *green arrows* positive regression coefficients. Shown are standardized parameter estimates (Model parameters in Table 1, and S4 Table).
(JPG)

S1 Table Bird traits as used in analysis. The traits have been replicated from Renner and Hoesel [34]. Metadata of the data set with detailed descriptor of the variables including unit (if applicable) and source.
(PDF)

S2 Table Selection of arthropods data for analysis. x: Selected for analysis;; ’Selection 1’ vs. ’Selection 2’ refers to the inclusion of parameters for iterative structural equation modeling (SEM) in relation to arthropod abundance. Reasons for exclusion of parameters are listed in the column ’Usefulness for SEM Analysis.’ For species number SEMs, Selection PT (pitfall traps) and Selection FIT (flight interception traps) of arthropods were merged and used.
(PDF)

S3 Table LiDAR parameters with brief description and metadata for LiDAR variables. The 10 were finally selected through Random Forest algorithm. The original dataset and metadata are archived through the Biodiversity Exploratories and not repeated here.
(PDF)

S4 Table Structural Equations Modeling (SEM) output of models. Response is abundance in all cases. Parameter Estimates and Standardized Solution are shown for each SEM. *block/group*: block and group in multigroup SEM; s.e. standard error. The table is attached as an extra excel file for the 14 SEM.
(XLSX)

S5 Table Piecewise Structural Equations Modeling (SEM) output of models X2 and Y1. Response is abundance in all cases. ***: *p* ≤ 0.001; ** *p* ≤ 0.01; * *p* ≤ 0.05.
(PDF)
