# Supplementary figures and images for: Forest structure, plants, arthropods, scale, or birds’ functional groups: What key factor are forest birds responding to?
Source: PLoS One. 2024 May 31;19(5):e0304421. doi: 10.1371/journal.pone.0304421 (PMC11142435; doi:10.1371/journal.pone.0304421)

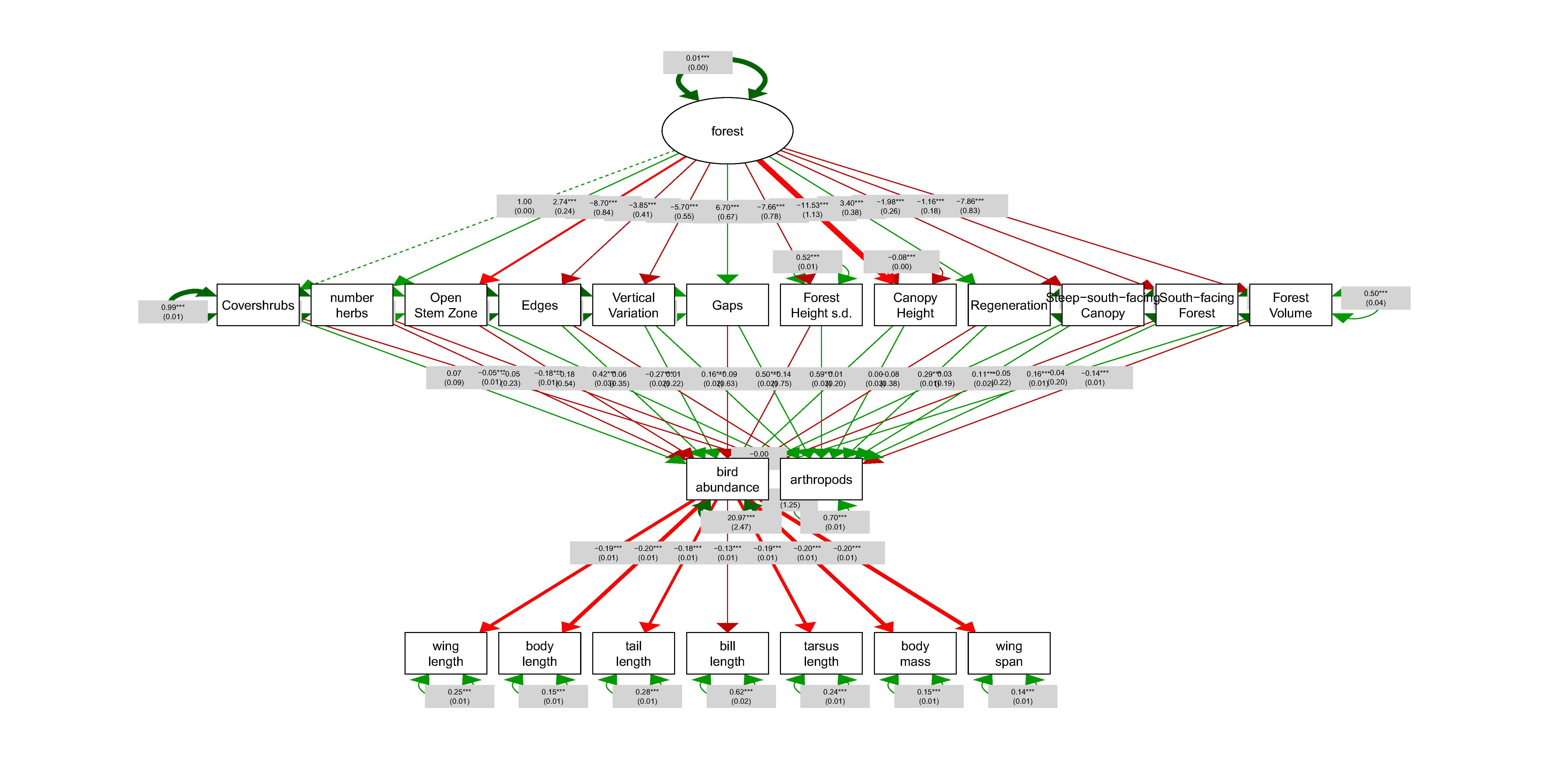

Supplement: S1 Fig — Double-headed or bidirectional arrows indicate variance or covariance. Latent variables (ellipse) are unobserved variables. Red arrows indicate negative vs. green arrows positive regression coefficient. Shown are standardized parameter estimates including s.e.; ***: p ≤ 0.001; ** p ≤ 0.01; * p ≤ 0.05. (Model parameters in Table 1, and S4 Table). (JPG) [file pone.0304421.s001.jpg]

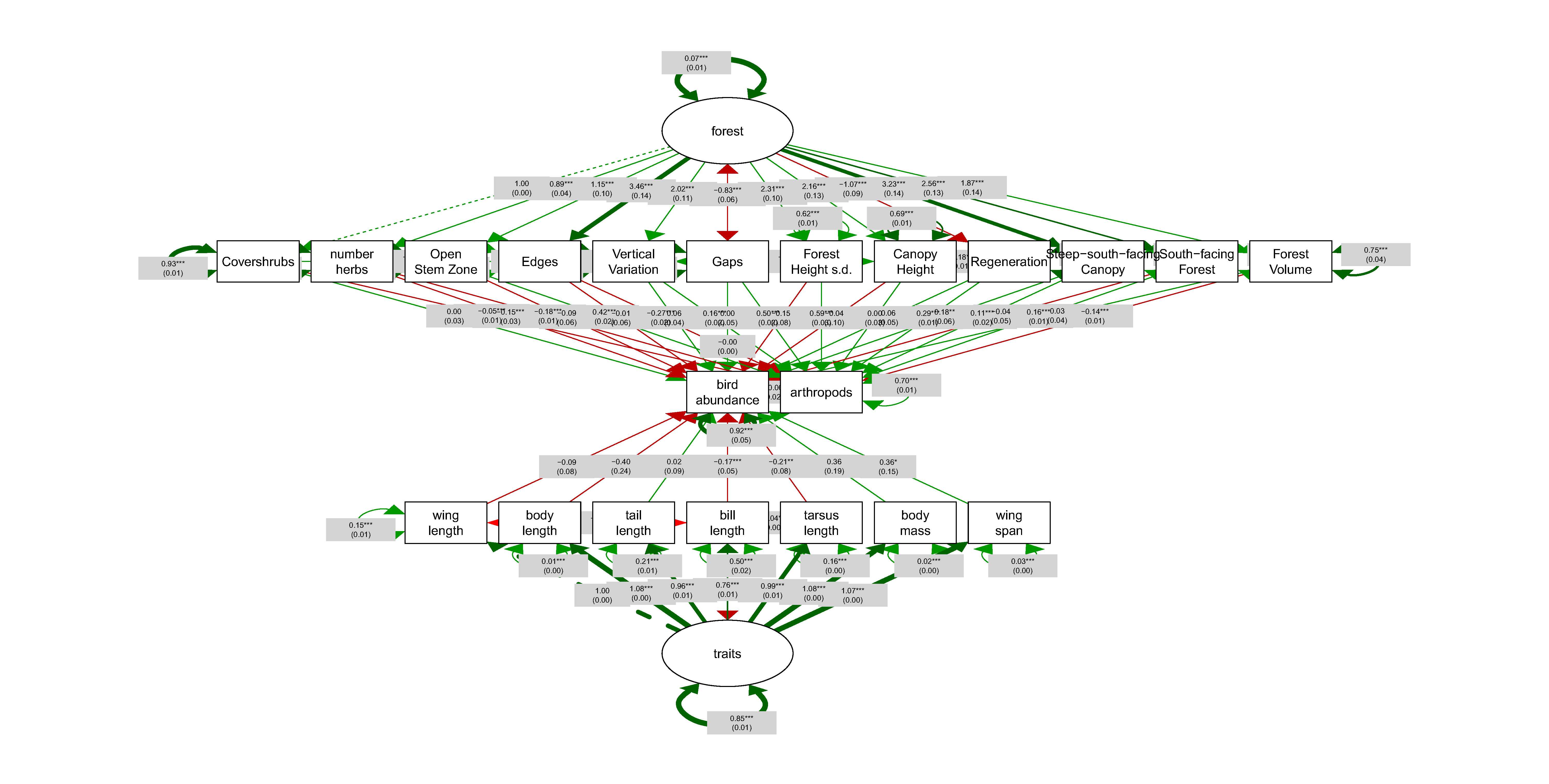

Supplement: S2 Fig — Double-headed or bidirectional arrows indicate variance or covariance. Latent variables (ellipse) are unobserved variables. Red arrows indicate negative vs. green arrows positive regression coefficients. Shown are standardized parameter estimates including s.e.; ***: p ≤ 0.001; ** p ≤ 0.01; * p ≤ 0.05. (Model parameters in Table 1 and S4 Table). (JPG) [file pone.0304421.s002.jpg]

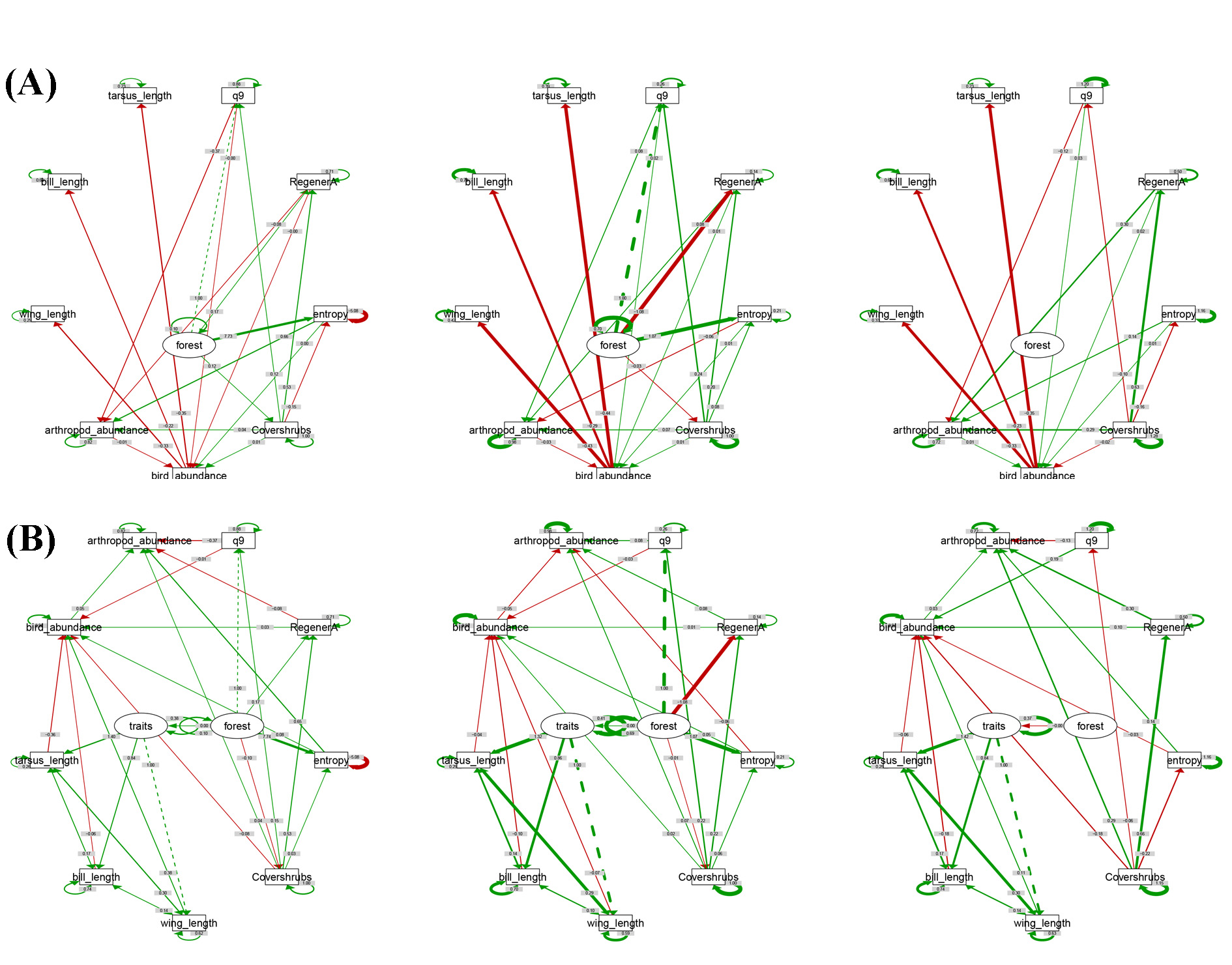

Supplement: S3 Fig — (A) Structural Equations Model "m1.fit.g3Expl" showing the SEM as for m1/H1, but grouped for the three regions. Shown are the SEM for each of the three Exploratories from left to right as: Schwäbische Alb (southwest), Hainich-Dün (center), and Schorfheide-Chorin (northeast). (B) Structural Equations Model "m2.fit.g3Expl" showing the SEM as for m2/H2, but grouped for the three regions (same sequence from left to right). Double-headed or bidirectional arrows indicate variance or covariance. Latent variables (ellipse) are unobserved variables. Red arrows indicate negative vs. green arrows positive regression coefficients. Shown are standardized parameter estimates (Model parameters in Table 1, and S4 Table). (JPG) [file pone.0304421.s003.jpg]

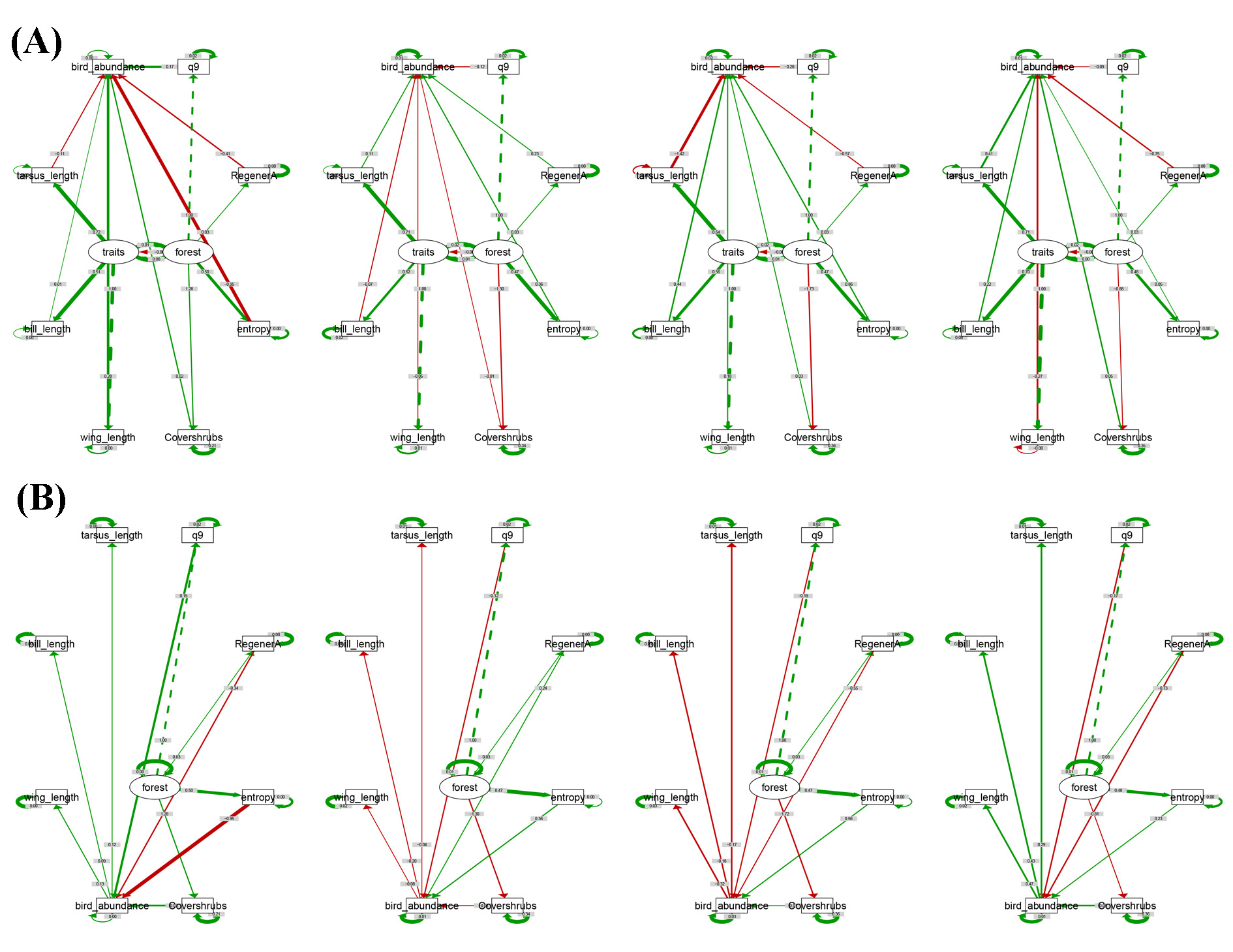

Supplement: S4 Fig — (A) Structural Equations Model "m3na.fg" showing the SEM as for m1/H1, but grouped for four functional groups. Shown are the SEM for each of the four functional groups from left to right as: carnivore, insectivore, granivore, and omnivore. (B) Structural Equations Model "m4na.fg" showing the SEM as for m2/H2, but grouped for four functional groups (same sequence from left to right). Double-headed or bidirectional arrows indicate variance or covariance. Latent variables (ellipse) are unobserved variables. Red arrows indicate negative vs. green arrows positive regression coefficients. Shown are standardized parameter estimates (Model parameters in Table 1, and S4 Table). (JPG) [file pone.0304421.s004.jpg]
