# Supplementary material for: Forest structure, plants, arthropods, scale, or birds’ functional groups: What key factor are forest birds responding to?
Source: PLoS One. 2024 May 31;19(5):e0304421. doi: 10.1371/journal.pone.0304421 (PMC11142435; doi:10.1371/journal.pone.0304421)
Supplement: S1 Table — The traits have been replicated from Renner and Hoesel [34]. Metadata of the data set with detailed descriptor of the variables including unit (if applicable) and source. (PDF) [file pone.0304421.s005.pdf]

Table S1. Bird traits as used in analysis. The traits have been replicated from Renner and Hoesel [34]. Metadata of the data set with detailed descriptor of the variables including unit (if applicable) and source.

Scientific name	English name	Functional group	Wing length	Body Length	Tail Length	Bill Length	Tarsus Length	Body Mass
Accipiter gentilis	Northern Goshawk	carnivore	360	62	270.0	24.0	78.0	1350
Acrocephalus palustris	Marsh Warbler	insectivore	76	14	61.0	17.2	19.4	15
Acrocephalus scirpaceus	Eurasian Reed-warbler	insectivore	73	14	56.2	18.5	24.1	15
Aegithalos caudatus	Long-tailed Tit	insectivore	64	15	92.0	7.0	17.5	9
Alauda arvensis	Eurasian Skylark	insectivore	118	19	74.0	16.3	27.0	45
Anser anser	Greylag Goose	herbivore	480	90	74.0	150.0	82.0	3700
Anthus pratensis	Meadow Pipit	insectivore	14.9	15	63.5	86.0	21.8	25
Anthus trivialis	Tree Pipit	insectivore	15.8	16	65.5	91.0	23.2	25
Apus apus	Common Swift	insectivore	187	17	85.0	14.0	13.0	50
Ardea cinerea	Grey Heron	carnivore	470	98	175.0	125.0	165.0	2000
Buteo buteo	Common Buzzard	carnivore	424	57	222.0	25.5	82.0	1300
Carduelis cannabina	Common Linnet	granivore	86	14	57.0	13.2	16.9	20
Carduelis carduelis	European Goldfinch	granivore	87	13	52.5	16.5	15.4	18
Carduelis chloris	Greenfinch	granivore	91	16	59.0	20.9	18.9	34
Carduelis spinus	Eurasian Siskin	granivore	77	12	48.0	14.1	14.6	14
Certhia brachydactyla	Short-toed Treecreeper	insectivore	67.5	13	64.5	23.0	17.2	12
Certhia familiaris	Eurasian Treecreeper	insectivore	67	14	70.0	21.2	17.0	12
Ciconia ciconia	White Stork	omnivore	630	115	240.0	190.0	240.0	3500
Coccothraustes coccothraustes	Hawfinch	granivore	112	18	54.0	22.6	23.0	62
Columba oenas	Stock Dove	granivore	226	34	115.0	21.0	32.0	340
Columba palumbus	Common Woodpigeon	granivore	258	42	185.0	23.5	35.0	520
Corvus corax	Common Raven	omnivore	442	67	246.0	84.0	73.5	1500
Corvus corone cornix	Hooded Crow	omnivore	335	51	219.0	58.0	62.0	600
Corvus corone corone	Carrion Crow	omnivore	345	51	203.0	63.0	63.4	600
Corvus monedula	Jackdaw	omnivore	247	34	139.0	36.5	46.0	270
Coturnix coturnix	Common Quail	granivore	119	18	43.0	13.0	27.5	135
Cuculus canorus	Cuckoo	insectivore	230	34	178.0	24.0	24.5	130
Cyanistes caeruleus	Blue Tit	insectivore	71	12	57.0	11.5	18.5	12
Delichon urbica	House Martin	insectivore	118	13	66.0	8.0	11.7	21
Dendrocopos major	Great-spotted Woodpecker	insectivore	150	23	91.5	31.5	24.9	90
Dendrocopos medius	Middle-spotted Woodpecker	insectivore	131	22	86.5	26.2	23.0	80
Dendrocopos minor	Lesser-spotted Woodpecker	insectivore	99	15	65.0	19.0	16.5	22
Dryocopus martius	Black Woodpecker	insectivore	241	46	173.0	62.0	39.0	350
Emberiza calandra	Corn Bunting	granivore	105	19	81.0	18.7	27.1	55
Emberiza citrinella	Yellowhammer	granivore	95	17	84.0	17.2	21.2	30
Erithacus rubecula	European Robin	insectivore	77	14	63.5	16.4	27.0	22
Falco tinnunculus	Common Kestrel	carnivore	271	32	188.0	17.0	47.2	300
Ficedula hypoleuca	Pied Flycatcher	insectivore	83	13	56.0	14.2	18.0	15
Ficedula parva	Red-breasted Flycatcher	insectivore	73	12	54.9	10.3	18.2	13
Fringilla coelebs	Chaffinch	granivore	92	16	73.4	15.0	23.0	24
Fringilla montifringilla	Brambling	granivore	96.5	16	70.0	17.8	20.3	29
Gallinago gallinago	Common Snipe	omnivore	140	28	61.0	75.0	36.5	120
Garrulus glandarius	Eurasian Jay	omnivore	195	35	163.0	42.0	45.3	190
Grus grus	Common Crane	omnivore	610	119	220.0	118.0	260.0	7000

Scientific name	English name	Functional group	Wing length	Body Length	Tail Length	Bill Length	Tarsus Length	Body Mass
Hippolais icterina	Icterine Warbler	insectivore	82	14	60.0	17.5	23.0	22
Hirundo rustica	Barn Swallow	insectivore	131	21	132.0	14.0	11.5	25
Jynx torquilla	Eurasian Wryneck	insectivore	93	17	67.0	17.3	20.5	45
Lanius collurio	Red backed shrike	insectivore	100	18	83.0	17.0	29.0	40
Locustella naevia	Common Grashopper-warbler	insectivore	68	13	59.0	13.5	21.0	15
Loxia curvirostra	Common Crossbill	granivore	103	18	61.0	21.2	21.0	40
Lullula arborea	Woodlark	insectivore	97	15	54.0	12.0	23.0	36
Luscinia megarhynchos	Common Nightingale	insectivore	86	17	68.0	18.2	29.4	27
Milvus migrans	Black Kite	carnivore	480	60	280.0	28.0	62.0	950
Milvus milvus	Red Kite	carnivore	535	73	390.0	29.0	61.0	1300
Motacilla alba	Pied Wagtail	insectivore	86	19	75.5	92.0	25.5	27
Motacilla flava	Yellow Wagtail	insectivore	94	18	90.0	76.0	25.0	22
Muscicapa striata	Spotted flycatcher	insectivore	94	15	65.0	17.8	16.0	19
Oenanthe oenanthe	Northern Weateater	insectivore	102	16	60.0	18.8	30.0	41
Oriolus oriolus	Golden Oriole	insectivore	163	25	94.5	26.5	24.0	67
Parus cristatus	Crested Tit	insectivore	67	12	56.3	8.5	19.0	13
Parus major	Great Tit	insectivore	81	15	71.5	16.0	20.8	21
Parus montanus	Willow Tit	insectivore	68	13	64.0	11.6	17.6	12
Parus palustris	Marsh Tit	insectivore	70	13	60.0	11.0	17.1	12
Passer domesticus	House Sparrow	granivore	83	15	60.0	15.5	19.7	32
Passer montanus	Tree Sparrow	granivore	72	14	57.0	14.6	18.3	25
Periparus ater	Coal Tit	insectivore	65	12	53.0	12.0	18.3	10
Pernis apivorus	European Honey-buzzard	insectivore	441	60	276.0	23.0	55.0	1100
Phasianus colchicus	Pheasant	omnivore	274	90	560.0	32.5	77.0	1400
Phoenicurus ochruros	Black Redstart	insectivore	91	15	65.0	16.3	24.1	19
Phoenicurus phoenicurus	Common Redstart	insectivore	85	15	61.5	15.8	23.3	20
Phylloscopus bonelli	Bonelli s warbler	insectivore	68	12	13.1	13.5	19.7	9
Phylloscopus collybita	Common chiffchaff	insectivore	68	12	54.0	12.5	21.0	9
Phylloscopus sibilatrix	Wood warbler	insectivore	81	13	56.0	13.6	22.0	12
Phylloscopus trochilus	Willow warbler	insectivore	70	12	13.7	13.0	21.0	10
Pica pica	Magpie	omnivore	215	51	296.0	44.6	52.6	250
Picus canus	Grey headed woodpecker	insectivore	155	26	104.0	44.0	28.5	165
Picus viridis	Green woodpecker	insectivore	171	33	104.0	53.0	33.0	220
Prunella modularis	Dunnock	insectivore	74	14	62.0	12.7	21.9	24
Pyrrhula pyrrhula	Bullfinch	granivore	95	16	73.0	15.9	18.4	27
Regulus ignicapillus	Firecrest	insectivore	56	10	44.0	14.6	21.2	7
Regulus regulus	Goldcrest	insectivore	58	10	59.0	8.0	19.0	7
Saxicola rubetra	Whinchat	insectivore	83	14	49.0	16.3	24.0	24
Saxicola rubicola	Stonechat	insectivore	71	13	52.0	15.5	24.5	17
Scolopax rusticola	Eurasian Woodcock	carnivore	214	35	90.0	80.0	40.0	420
Sitta europaea	Eurasian nuthatch	insectivore	92	15	49.0	21.8	20.7	24
Streptopelia turtur	European Turtle Dove	granivore	182	28	112.0	19.0	24.0	180
Strix aluco	Tawny owl	carnivore	298	39	185.0	29.5	53.0	590
Sturnus vulgaris	European starling	insectivore	138	22	66.0	29.5	30.3	90
Sylvia atricapilla	Blackcap	insectivore	80	15	62.0	16.1	21.5	20
Sylvia borin	Garden warbler	insectivore	83	14	58.0	15.1	21.8	23
Sylvia communis	Common whitethroat	insectivore	77	15	67.0	57.4	22.5	18
Sylvia curucca	Lesser whitethroat	insectivore	70	14	58.0	13.9	20.4	16

**Scientific name**	**English name**	**Functional group**	**Wing length**	**Body Length**	**Tail Length**	**Bill Length**	**Tarsus Length**	**Body Mass**
Sylvia nisoria	Barred Warbler	insectivore	93	17	79.0	18.3	28.0	35
Troglodytes troglodytes	Winter wren	insectivore	52	10	35.0	12.2	18.5	13
Turdus iliacus	Redwing	insectivore	127	23	89.0	23.5	32.0	75
Turdus merula	Common blackbird	insectivore	138	29	116.0	32.5	35.0	110
Turdus philomelos	Song thrush	insectivore	123	24	90.0	24.0	33.8	90
Turdus pilaris	Fieldfare	insectivore	153	27	115.0	25.7	34.4	140
Turdus viscivorus	Mistle thrush	insectivore	164	29	118.0	26.5	35.0	140
Vanellus vanellus	Lapwing	insectivore	n/a	n/a	n/a	n/a	n/a	n/a
