# Supplementary material for: Forest structure, plants, arthropods, scale, or birds’ functional groups: What key factor are forest birds responding to?
Source: PLoS One. 2024 May 31;19(5):e0304421. doi: 10.1371/journal.pone.0304421 (PMC11142435; doi:10.1371/journal.pone.0304421)
Supplement: S2 Table — x: Selected for analysis;; ’Selection 1’ vs. ’Selection 2’ refers to the inclusion of parameters for iterative structural equation modeling (SEM) in relation to arthropod abundance. Reasons for exclusion of parameters are listed in the column ’Usefulness for SEM Analysis.’ For species number SEMs, Selection PT (pitfall traps) and Selection FIT (flight interception traps) of arthropods were merged and used. (PDF) [file pone.0304421.s006.pdf]

Table S2. Selection of arthropods data for analysis. x: Selected for analysis; ; 'Selection 1' vs. 'Selection 2' refers to the inclusion of parameters for iterative structural equation modeling (SEM) in relation to arthropod abundance. Reasons for exclusion of parameters are listed in the column 'Usefulness for SEM Analysis.' For species number SEMs, Selection PT (pitfall traps) and Selection FIT (flight interception traps) of arthropods were merged and used.

**Taxon**		**Usefulness for SEM analysis**	**Selection 1**	**Selection 2**	**Selection PT**	**Selection FIT**
			Abundance		Species numbers	
Arachnida						
	Acari	No - sampling not representative				
	Araneae	Yes	x	x	x	
	Opiliones	Yes	x	x	x	
	Pseudoscorpiones	Yes	x	x	x	
Chilopoda		Yes	x	x		
Diplopoda		Yes	x	x		
Malacostraca	Isopoda	Yes	x	x		
Clitellata	Lumbricidae	Yes	x	x		
Gastropoda		Yes	x	x		
Insecta						
Coleoptera	Carabidae	Yes	x	x	x	x
	Coleoptera-other	Yes, but relatively large group	x	x	x	x
	Staphylinidae	Yes	x	x	x	x
Collembola		No				
Dermaptera		Yes	x	x	x	x
Dictyoptera	Blattodea	Yes	x	x	x	x
Diptera	Brachycera	Yes	x	x		
	Nematocera	Yes	x	x		
Ephemeroptera		Yes	x	x		
Hemiptera	Auchenorrhyncha	Yes	x	x	x	x
	Heteroptera	Yes	x	x	x	x
	Psyllidae	Yes	x	x		
	Sternorrhyncha	Yes	x	x		
Hymenoptera	Apocrita-other	No - Variation in dataset is too large		x		
	Formicidae	Yes	x	x		
	Symphyta	Yes	x	x	x	x
Lepidoptera		Yes	x	x		
Mecoptera		Yes	x	x	x	x
Megaloptera		Yes	x	x		
Neuroptera		Yes	x	x	x	x
Odonata		Yes	x	x		
Orthoptera	Caelifera	Yes	x	x	x	x
	Ensifera	Yes	x	x	x	x
Plecoptera		Yes	x	x		
Psocoptera		Yes	x	x		
Raphidioptera		Yes	x	x	x	x
Siphonaptera		No				
Thysanoptera		Yes	x	x		
Trichoptera		Yes	x	x		
	Larvae - holometabolic	Yes	x	x		
	Other	No				
Vertebrates						
	Muridae/Soricidae	No - sampling not representative		x		
Reptilia		Yes - important for carnivore birds, but sampling not representative		x		
Amphibia		No - sampling not representative		x		
