# Supplementary material for: Forest structure, plants, arthropods, scale, or birds’ functional groups: What key factor are forest birds responding to?
Source: PLoS One. 2024 May 31;19(5):e0304421. doi: 10.1371/journal.pone.0304421 (PMC11142435; doi:10.1371/journal.pone.0304421)
Supplement: S3 Table — The 10 were finally selected through Random Forest algorithm. The original dataset and metadata are archived through the Biodiversity Exploratories and not repeated here. (PDF) [file pone.0304421.s007.pdf]

Table S3. LiDAR parameters with brief description and Metadata for LiDAR variables. The 10 were finally selected through Random Forest algorithm. The original dataset and metadata are archived through the Biodiversity Exploratories and not repeated here.

LiDAR selected for study	Original variable name	Description
Open Stem Zone	bole Zone3	bole zone, but omitting values of deep gaps more than 3 m²
Edges	edgeN3	the real amount of distinct areas, that constitute forest edges, omitting areas less than 3 m²
Vertical Variation	entropy	the local vertical variation that is high for equal distributed echoes, low for clustered echoes; it is a parameter of texture
Gaps	gapN1	the real amount of distinct areas, that are gaps, omitting areas less than 1 m²
Forest Height s. d.	lochSD	the variation of the outer canopy height
Canopy Height	q9	the 0.9 quantile of the height of the outer canopy surface as a more robust measure
Regeneration-layer	RegenerA	the relative amount of secured regeneration from 1.5m to 2.5m
Steep-south-facing Canopy	southFraction1	the relative amount of projected level of ground area where the outer canopy surface has a south aspect and a comparatively steep slope, omitting areas less than 1 m²
South-facing Forest	southN3	the real amount of distinct areas, that are south facing, omitting areas less than 3 m²
Forest Volume	Volume	the amount of wood in the forest. Values of height less than 10m are omitted
