# Supplementary material for: Forest structure, plants, arthropods, scale, or birds’ functional groups: What key factor are forest birds responding to?
Source: PLoS One. 2024 May 31;19(5):e0304421. doi: 10.1371/journal.pone.0304421 (PMC11142435; doi:10.1371/journal.pone.0304421)
Supplement: S5 Table — Response is abundance in all cases. ***: p ≤ 0.001; ** p ≤ 0.01; * p ≤ 0.05. (PDF) [file pone.0304421.s009.pdf]

Table S5. Piecewise Structural Equations Modeling (SEM) output of models X2 and Y1. Response is abundance in all cases. ***: $p \leq 0.001$; ** $p \leq 0.01$; * $p \leq 0.05$.

X2								
**Tests of directed separation**								
Independent Claim	Test type			DF	Critical value	*p*		
arthropod abundance ~ wing length + ...	coef			1466	-1.113	0.266		
arthropod abundance ~ body length + ...	coef			1466	-0.966	0.334		
arthropod abundance ~ tail length + ...	coef			1466	-1.562	0.119		
arthropod abundance ~ bill length + ...	coef			1466	-0.772	0.440		
arthropod abundance ~ tarsus length + ...	coef			1466	-0.948	0.343		
arthropod abundance ~ body mass + ...	coef			1466	-0.758	0.448		
arthropod abundance ~ wing span + ...	coef			1466	-0.702	0.483		
**Coefficients**								
Response	Predictor	Estimate	s.e.	DF	Critical value	*p*	Std. Estimate	
bird abundance	Covershrubs	0.001	0.006	1461	0.119	0.905	0.004	
bird abundance	number herbs	-0.049	0.010	1461	-4.791	0	-0.155	***
bird abundance	boleZone3	-0.052	0.038	1461	-1.383	0.167	-0.095	
bird abundance	edgeN3	-0.002	0.012	1461	-0.137	0.891	-0.007	
bird abundance	entropy	0.160	0.107	1461	1.495	0.135	0.065	
bird abundance	gapN1	0.003	0.031	1461	0.087	0.930	0.005	
bird abundance	lochSD	-0.089	0.048	1461	-1.870	0.062	-0.15	
bird abundance	q9	-0.029	0.068	1461	-0.426	0.670	-0.043	
bird abundance	RegenerA	0.161	0.132	1461	1.219	0.223	0.058	
bird abundance	southFraction1	1.006	0.382	1461	2.633	0.009	0.175	**
bird abundance	southN3	-0.018	0.026	1461	-0.699	0.485	-0.042	
bird abundance	Volumen	0.005	0.008	1461	0.623	0.533	0.029	
bird abundance	wing length	-0.038	0.053	1461	-0.718	0.473	-0.089	
bird abundance	body length	-0.227	0.142	1461	-1.590	0.112	-0.404	
bird abundance	tail length	0.011	0.040	1461	0.275	0.784	0.024	
bird abundance	bill length	-0.088	0.029	1461	-3.014	0.003	-0.172	**
bird abundance	tarsus length	-0.138	0.052	1461	-2.659	0.008	-0.208	**
bird abundance	body mass	0.067	0.039	1461	1.696	0.09	0.358	
bird abundance	wing span	0.171	0.079	1461	2.159	0.031	0.363	*
arthropod abundance	bird abundance	0.005	0.037	1467	0.129	0.898	0.003	
arthropod abundance	Covershrubs	-0.016	0.009	1467	-1.805	0.071	-0.05	
arthropod abundance	number herbs	-0.100	0.015	1467	-6.885	0	-0.191	***
arthropod abundance	boleZone3	0.412	0.054	1467	7.679	0	0.430	***
arthropod abundance	edgeN3	-0.077	0.018	1467	-4.377	0	-0.197	***
arthropod abundance	entropy	0.295	0.153	1467	1.929	0.0539	0.072	
arthropod abundance	gapN1	0.398	0.045	1467	8.88	0	0.399	***
arthropod abundance	lochSD	0.525	0.068	1467	7.708	0	0.541	***
arthropod abundance	q9	0.025	0.097	1467	0.253	0.800	0.022	
arthropod abundance	RegenerA	1.312	0.188	1467	6.968	0	0.285	***
arthropod abundance	southFraction1	1.376	0.548	1467	2.512	0.012	0.144	*
arthropod abundance	southN3	0.123	0.037	1467	3.318	0.0009	0.170	***
arthropod abundance	Volumen	-0.051	0.012	1467	-4.197	0	-0.168	***
**Individual R^2**	**Response method**	**R^2**						
bird abundance	none	0.08						
arthropod abundance	none	0.32						
Y1								
**Tests of directed separation**								
Independ.Claim	Test type			DF	Critical value	*p*		
bill length ~ Covershrubs + ...	coef			1478	2.188	0.029		*
body length ~ Covershrubs + ...	coef			1478	3.045	0.002		**
body mass ~ Covershrubs + ...	coef			1478	3.097	0.002		**

tail length ~ Covershrubs + ...	coef	1478	2.090	0.036	*
tarsus length ~ Covershrubs + ...	coef	1478	3.280	0.001	**
wing length ~ Covershrubs + ...	coef	1478	2.257	0.024	*
wing span ~ Covershrubs + ...	coef	1478	2.894	0.004	**
bill length ~ number herbs + ...	coef	1478	-0.929	0.353	
body length ~ number herbs + ...	coef	1478	-0.291	0.771	
body mass ~ number herbs + ...	coef	1478	-0.049	0.961	
tail length ~ number herbs + ...	coef	1478	0.208	0.835	
tarsus length ~ number herbs + ...	coef	1478	0.895	0.371	
wing length ~ number herbs + ...	coef	1478	-0.376	0.707	
wing span ~ number herbs + ...	coef	1478	-1.042	0.298	
bill length ~ boleZone3 + ...	coef	1478	-2.050	0.042	*
body length ~ boleZone3 + ...	coef	1478	-3.929	0.001	***
body mass ~ boleZone3 + ...	coef	1478	-3.521	0.001	***
tail length ~ boleZone3 + ...	coef	1478	-2.557	0.011	*
tarsus length ~ boleZone3 + ...	coef	1478	-4.036	0.001	***
wing length ~ boleZone3 + ...	coef	1478	-3.959	0.001	***
wing span ~ boleZone3 + ...	coef	1478	-3.511	0.001	***
bill length ~ edgeN3 + ...	coef	1478	0.682	0.495	
body length ~ edgeN3 + ...	coef	1478	-0.061	0.952	
body mass ~ edgeN3 + ...	coef	1478	0.451	0.652	
tail length ~ edgeN3 + ...	coef	1478	-0.023	0.982	
tarsus length ~ edgeN3 + ...	coef	1478	-0.422	0.673	
wing length ~ edgeN3 + ...	coef	1478	-0.343	0.732	
wing span ~ edgeN3 + ...	coef	1478	0.221	0.825	
bill length ~ entropy + ...	coef	1478	0.284	0.777	
body length ~ entropy + ...	coef	1478	0.333	0.740	
body mass ~ entropy + ...	coef	1478	0.836	0.403	
tail length ~ entropy + ...	coef	1478	1.139	0.255	
tarsus length ~ entropy + ...	coef	1478	-0.414	0.679	
wing length ~ entropy + ...	coef	1478	0.747	0.455	
wing span ~ entropy + ...	coef	1478	0.869	0.385	
bill length ~ gapN1 + ...	coef	1478	-0.242	0.809	
body length ~ gapN1 + ...	coef	1478	0.635	0.526	
body mass ~ gapN1 + ...	coef	1478	0.264	0.792	
tail length ~ gapN1 + ...	coef	1478	1.415	0.157	
tarsus length ~ gapN1 + ...	coef	1478	1.026	0.305	
wing length ~ gapN1 + ...	coef	1478	0.858	0.391	
wing span ~ gapN1 + ...	coef	1478	-0.053	0.958	
bill length ~ lochSD + ...	coef	1478	-0.718	0.473	
body length ~ lochSD + ...	coef	1478	-2.071	0.039	*
body mass ~ lochSD + ...	coef	1478	-1.593	0.111	
tail length ~ lochSD + ...	coef	1478	-2.101	0.036	*
tarsus length ~ lochSD + ...	coef	1478	-2.219	0.027	*
wing length ~ lochSD + ...	coef	1478	-2.736	0.006	**
wing span ~ lochSD + ...	coef	1478	-1.611	0.107	
bill length ~ q9 + ...	coef	1478	-1.240	0.215	
body length ~ q9 + ...	coef	1478	-2.778	0.006	**
body mass ~ q9 + ...	coef	1478	-2.208	0.027	*
tail length ~ q9 + ...	coef	1478	-1.888	0.059	
tarsus length ~ q9 + ...	coef	1478	-3.245	0.001	**
wing length ~ q9 + ...	coef	1478	-2.984	0.003	**
wing span ~ q9 + ...	coef	1478	-2.135	0.033	*
bill length ~ RegenerA + ...	coef	1478	-0.122	0.903	
body length ~ RegenerA + ...	coef	1478	0.621	0.535	
body mass ~ RegenerA + ...	coef	1478	0.347	0.729	
tail length ~ RegenerA + ...	coef	1478	-0.462	0.644	
tarsus length ~ RegenerA + ...	coef	1478	0.880	0.379	
wing length ~ RegenerA + ...	coef	1478	0.376	0.707	
wing span ~ RegenerA + ...	coef	1478	0.395	0.693	
bill length ~ southFraction1 + ...	coef	1478	0.709	0.478	
body length ~ southFraction1 + ...	coef	1478	-0.114	0.909	

body mass ~ southFraction1 + ...	coef			1478	0.328	0.743		
tail length ~ southFraction1 + ...	coef			1478	0.149	0.881		
tarsus length ~ southFraction1 + ...	coef			1478	-0.066	0.948		
wing length ~ southFraction1 + ...	coef			1478	-0.586	0.558		
wing span ~ southFraction1 + ...	coef			1478	0.033	0.974		
bill length ~ southN3 + ...	coef			1478	-0.795	0.427		
body length ~ southN3 + ...	coef			1478	-1.620	0.105		
body mass ~ southN3 + ...	coef			1478	-1.268	0.205		
tail length ~ southN3 + ...	coef			1478	-0.804	0.422		
tarsus length ~ southN3 + ...	coef			1478	-1.556	0.120		
wing length ~ southN3 + ...	coef			1478	-1.805	0.071		
wing span ~ southN3 + ...	coef			1478	-1.622	0.105		
bill length ~ Volumen + ...	coef			1478	-0.277	0.782		
body length ~ Volumen + ...	coef			1478	-0.923	0.356		
body mass ~ Volumen + ...	coef			1478	-0.442	0.658		
tail length ~ Volumen + ...	coef			1478	0.162	0.871		
tarsus length ~ Volumen + ...	coef			1478	-1.702	0.089		
wing length ~ Volumen + ...	coef			1478	-0.994	0.321		
wing span ~ Volumen + ...	coef			1478	-0.572	0.568		
bill length ~ arthropod abundance + ...	coef			1466	-0.772	0.440		
body length ~ arthropod abundance + ...	coef			1466	-0.966	0.334		
body mass ~ arthropod abundance + ...	coef			1466	-0.758	0.448		
tail length ~ arthropod abundance + ...	coef			1466	-1.562	0.119		
tarsus length ~ arthropod abundance + ...	coef			1466	-0.948	0.343		
wing length ~ arthropod abundance + ...	coef			1466	-1.113	0.266		
wing span ~ arthropod abundance + ...	coef			1466	-0.702	0.483		
body length ~ bill length + ...	coef			1478	45.145	0		***
body mass ~ bill length + ...	coef			1478	46.100	0		***
tail length ~ bill length + ...	coef			1478	31.4254	0		***
tarsus length ~ bill length + ...	coef			1478	44.091	0		***
wing length ~ bill length + ...	coef			1478	28.088	0		***
wing span ~ bill length + ...	coef			1478	42.770	0		***
body mass ~ body length + ...	coef			1478	207.753	0		***
tail length ~ body length + ...	coef			1478	82.483	0		***
tarsus length ~ body length + ...	coef			1478	67.959	0		***
wing length ~ body length + ...	coef			1478	101.243	0		***
wing span ~ body length + ...	coef			1478	162.862	0		***
tail length ~ body mass + ...	coef			1478	67.175	0		***
tarsus length ~ body mass + ...	coef			1478	70.280	0		***
wing length ~ body mass + ...	coef			1478	92.631	0		***
wing span ~ body mass + ...	coef			1478	154.518	0		***
tarsus length ~ tail length + ...	coef			1478	50.110	0		***
wing length ~ tail length + ...	coef			1478	62.100	0		***
wing span ~ tail length + ...	coef			1478	67.092	0		***
wing length ~ tarsus length + ...	coef			1478	52.264	0		***
wing span ~ tarsus length + ...	coef			1478	59.278	0		***
wing span ~ wing length + ...	coef			1478	96.206	0		***

Coefficients

Response	Predictor	Estimate	s.e.	DF	Critical value	*p*	Std. Estimate	
bird abundance	Covershrubs	-0.001	0.006	1468	-0.135	0.892	-0.005	
bird abundance	number herbs	-0.050	0.010	1468	-4.889	0	-0.158	***
bird abundance	boleZone3	-0.048	0.038	1468	-1.265	0.206	-0.084	
bird abundance	edgeN3	-0.002	0.012	1468	-0.177	0.860	-0.009	
bird abundance	entropy	0.182	0.108	1468	1.687	0.092	0.074	
bird abundance	gapN1	0.005	0.032	1468	0.145	0.885	0.008	
bird abundance	lochSD	-0.084	0.048	1468	-1.750	0.080	-0.145	
bird abundance	q9	-0.030	0.068	1468	-0.443	0.658	-0.046	
bird abundance	RegenerA	0.191	0.133	1468	1.437	0.151	0.069	
bird abundance	southFraction1	0.966	0.387	1468	2.498	0.013	0.168	*
bird abundance	southN3	-0.013	0.026	1468	-0.503	0.615	-0.030	
bird abundance	Volumen	0.006	0.009	1468	0.673	0.501	0.032	

arthropod abundance	bird abundance	0.005	0.037	1467	0.129	0.898	0.003	
arthropod abundance	Covershrubs	-0.016	0.009	1467	-1.805	0.071	-0.052	
arthropod abundance	number herbs	-0.100	0.015	1467	-6.885	0	-0.191	***
arthropod abundance	boleZone3	0.412	0.054	1467	7.680	0	0.430	***
arthropod abundance	edgeN3	-0.077	0.018	1467	-4.377	0	-0.197	***
arthropod abundance	entropy	0.295	0.153	1467	1.929	0.054	0.072	
arthropod abundance	gapN1	0.398	0.045	1467	8.882	0	0.399	***
arthropod abundance	lochSD	0.525	0.068	1467	7.708	0	0.541	***
arthropod abundance	q9	0.025	0.100	1467	0.253	0.800	0.022	
arthropod abundance	RegenerA	1.312	0.188	1467	6.968	0	0.285	***
arthropod abundance	southFraction1	1.376	0.548	1467	2.512	0.012	0.144	*
arthropod abundance	southN3	0.123	0.037	1467	3.318	0.001	0.170	***
arthropod abundance	Volumen	-0.051	0.012	1467	-4.197	0	-0.170	***
bill length	bird abundance	-0.211	0.045	1479	-4.710	0	-0.108	***
body length	bird abundance	-0.093	0.036	1479	-2.547	0.011	-0.052	*
body mass	bird abundance	-0.247	0.106	1479	-2.339	0.020	-0.046	*
tail length	bird abundance	-0.100	0.042	1479	-2.391	0.017	-0.046	*
tarsus length	bird abundance	-0.118	0.028	1479	-4.192	0	-0.079	***
wing length	bird abundance	-0.065	0.044	1479	-1.505	0.133	-0.028	
wing span	bird abundance	-0.068	0.040	1479	-1.717	0.086	-0.032	

**Individual R^2**	**Response method**	**R^2**
bird abundance	none	0.05
arthropod abundance	none	0.32
bill length	none	0.01
body length	none	0
body mass	none	0
tail length	none	0
tarsus length	none	0.01
wing length	none	0
wing span	none	0

Piecewise SEM "X2" with the following structure: bird abundance ~ Covershrubs + number herbs + boleZone3 + edgeN3 + entropy + gapN1 + lochSD + q9 + RegenerA + southFraction1 + southN3 + Volumen + wing length + body length + tail length + bill length + tarsus length + body mass + wing span; arthropod abundance ~ bird abundance + Covershrubs + number herbs + boleZone3 + edgeN3 + entropy + gapN1 + lochSD + q9 + RegenerA + southFraction1 + southN3 + Volumen

Piecewise SEM "Y1" with the following structure: bird abundance ~ Covershrubs + number herbs + boleZone3 + edgeN3 + entropy + gapN1 + lochSD + q9 + RegenerA + southFraction1 + southN3 + Volumen;
arthropod abundance ~ bird abundance + Covershrubs + number herbs + boleZone3 + edgeN3 + entropy + gapN1 + lochSD + q9 + RegenerA + southFraction1 + southN3 + Volumen; bill length ~ bird abundance; body length ~ bird abundance; body mass ~ bird abundance; tail length ~ bird abundance; tarsus length ~ bird abundance; wing length ~ bird abundance; wing span ~ bird abundance;
